# Epiphytic Bacterial Community Analysis of *Ulva prolifera* in Garorim and Muan Bays, Republic of Korea

**DOI:** 10.3390/microorganisms12061142

**Published:** 2024-06-04

**Authors:** Mst Shamim Ara Supty, Kifat Jahan, Jun-Seok Lee, Keun-Hyung Choi

**Affiliations:** Department of Earth, Environmental and Space Sciences, Chungnam National University, Daejeon 34134, Republic of Korea

**Keywords:** bacterial community diversity, 16S rRNA gene amplicon sequencing, *Ulva prolifera*

## Abstract

The bacterial communities related to seaweed can vary considerably across different locations, and these variations influence the seaweed’s nutrition, growth, and development. To study this further, we evaluated the bacteria found on the green marine seaweed *Ulva prolifera* from Garorim Bay and Muan Bay, two key locations on Republic of Korea’s west coast. Our analysis found notable differences in the bacterial communities between the two locations. Garorim Bay hosted a more diverse bacterial population, with the highest number of ASVs (871) compared to Muan Bay’s 156 ASVs. In Muan Bay, more than 50% of the bacterial community was dominated by Pseudomonadota. On the other hand, Garorim Bay had a more balanced distribution between Bacteroidota and Pseudomonadota (37% and 35.5%, respectively). Additionally, Cyanobacteria, particularly *Cyanothece aeruginosa*, were found in significant numbers in Garorim Bay, making up 8% of the community. Mineral analysis indicated that Garorim Bay had higher levels of S, Na, Mg, Ca, and Fe. Function-wise, both locations exhibited bacterial enrichment in amino acid production, nucleosides, and nucleotide pathways. In conclusion, this study broadens our understanding of the bacterial communities associated with *Ulva prolifera* in Korean waters and provides a foundation for future research on the relationships between *U. prolifera* and its bacteria.

## 1. Introduction

**Figure 1:**
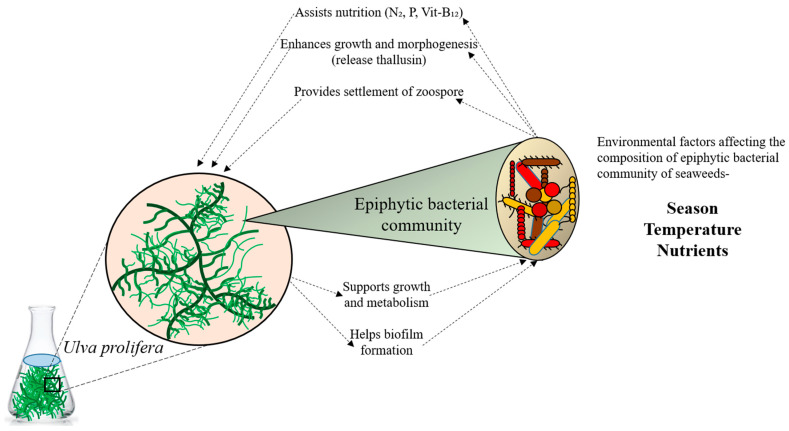
Relationship between seaweed and its epiphytic bacterial communities.

Bacteria inhabit all conceivable environments on Earth, performing various unique functions [1]. They influence both animal and plant development, as well as photosynthesis [2]. Moreover, bacteria establish symbiotic relationships with their hosts (Figure 1). The marine environment, covering the majority of the Earth’s surface, harbors diverse and intricate microbial communities [3]. Marine bacteria are indispensable in nutrient and organic carbon cycling [4]. Given their roles in primary production and as habitats for various animals, macroalgae also significantly impact the hard bottoms of tidal and subtidal zones in coastal marine ecosystems. While planktonic communities have traditionally been the focus of marine diversity surveys, there is growing interest in the microbiomes of eukaryotic hosts [5]. It is increasingly apparent that numerous marine eukaryotes maintain consistent relationships with their associated bacteria, relying on them for growth, development, and nutrition supply, as well as for protection from colonization and predation [5,6,7,8]. Macroalgae and bacteria also interact to supply nitrogen and macronutrients, such as vitamins and iron. In the microbial realm, where the distinction between host and symbiont is less clear, identifying partners in more complex communities and determining the benefits obtained by each can be challenging [9]. Advances in community modeling, extensive sampling of microbe-dominated biomes, and the emergence of metagenomics and other omics techniques all contribute to a paradigm shift in our comprehension of microbial interactions [10]. Currently, these methods are being employed to elucidate the interactions between bacteria and algae.

Research on the interactions of bacterial communities associated with seaweeds has significantly advanced in recent years [11,12]. This interaction may play a crucial role in tropical reef algae, suggesting an algal holobiont [5]. As our understanding continues to deepen, many specific aspects of these interactions have been identified [8,13,14,15]. These include the diversity of bacteria [15], the chemical interactions between seaweed and bacteria [8,14], and the microbial diseases that affect algae [13]. Consistent with prior findings, Pseudomonadota, Actinobacteria, Bacteroidota, and Acidobacteria have been revealed as vital for the cycling of carbon, nitrogen, and other nutrients. Alphaproteobacteria and Gammaproteobacteria are prevalent in oceanic and coastal waters [16,17], while Bacteroidota, Actinobacteria, Planctomycetes, and Chloroflexi are frequently observed as marine bacteria [16,18,19]. Certain green macroalgae require specific bacterial species for normal growth and morphology [20,21,22,23]. A recent review on *Ulva* demonstrated that two bacteria derived from *U. mutabilis* could induce morphogenesis and establish a tripartite community [24]. Additionally, it has been shown that algal-associated bacteria stimulate algal spore release and settlement, positively influence algal growth, provide essential nutrients, and promote the settlement of sea urchins and other larvae [25,26,27,28].

Metagenome technologies have effectively analyzed complex bacterial communities, enhancing the understanding of their functions, biotechnology, protein families, and ecology [29,30,31]. However, comprehensive examinations of all bacterial populations on algal surfaces are scarce [32], even though such knowledge is crucial to understanding seaweed–bacterial interactions and their role in coastal ecosystems. Geographical locations and physicochemical properties influence the distribution of these microbial communities. Fingerprinting studies involving denaturing gradient gel electrophoresis (DGGE) and 16S rRNA gene sequencing have demonstrated that these algal-associated communities differ from those on other living surfaces [14,33,34,35] and the surrounding saltwater [36].

Recent research by Dusedau et al. (2023) used 16S rRNA gene amplicon sequencing to explore the interaction between the red alga *Gracilaria vermiculophylla* and its epiphytic microbiome [37]. Burke et al. (2011) implemented metagenomic analysis of *U. australis*-associated bacterial communities and found that most sequences belonged to Pseudomonadota, Bacteroidota, and Planctomycetes [35]. The functional metagenomics of marine sponges and seaweed microbiomes have also disclosed new classes of antibacterial proteins [38]. Employing genome binning techniques, metagenomics can provide extensive inventories of a community’s metabolic and functional capacities, linking specific microbial species to ecosystem processes [39].

The edible green alga *Ulva prolifera* O.F. Müller is commonly found growing in the bays, river mouths, and intertidal flats of Korea [40]. It is primarily mass-produced in two significant locations, Muan and Garorim Bays, on the Korean west coast. This marine green macroalga often hosts a diverse microbiome. Our analysis focuses on the bacterial communities living on *Ulva prolifera* in Garorim Bay and Muan Bay. The study aims to uncover variations in community composition between these two distinctly different habitats.

## 2. Materials and Methods

### 2.1. Sample Collection and Preparation

**Figure 2:**
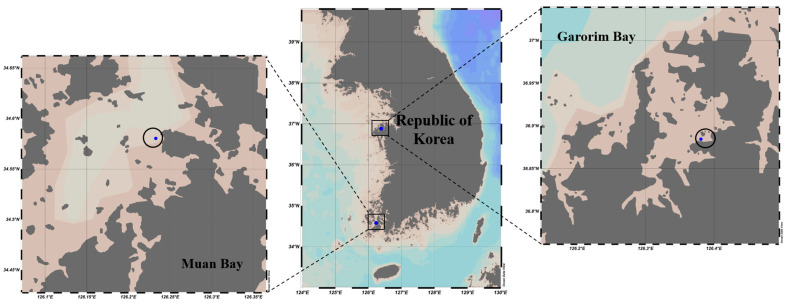
Two sampling locations of the seaweed *Ulva prolifera* (Garorim Bay and Muan Bay). Blue dots at each site indicate the sampling areas. These maps were created using Ocean Data View (v5.7.0).

In December 2022, we gathered 500 g of *Ulva prolifera* from Garorim Bay (36.884541 N, 126.380798 E) and 200 g from Muan Bay (34.99014 N, 126.47899 E). Two replications from each sampling location, Garorim Bay (Garorim 1, Garorim 2) and Muan Bay (Muan 1, Muan 2), were sampled (Figure 2). Around 100 m of sampling distance was maintained between the two replications of each bay. High fluctuations in salinity, ranging between 15.3 and 32.2 PSU, characterize Muan Bay. This volatility can be attributed to freshwater discharge from the Yeongsan River [41] potentially causing periodic fluctuations in nutrient levels. In contrast, Garorim Bay exhibits a high but stable salinity, fluctuating minutely from 30.1 PSU (in October) to 32.19 PSU (in February) [42]. This bay has a narrow basin and very limited freshwater inflow because of the absence of a major river. Algae samples were promptly transported at low temperatures to the lab in sterile plastic bags, where they were washed three times with sterilized seawater to eliminate debris and sediment. For molecular examination, samples of algae from each location were preserved at −80 °C.

### 2.2. DNA Extraction and Quantification

We extracted the DNA of epiphytic microbes for metagenomic analysis from seaweed following the developed DNA extraction method for *Ulva australis* epiphytic microbiome by Burke et al. (2009) [18]. The algal samples (10 g) exhibited a nearly complete and reproducible removal of the surface community following incubation in a 50 mL buffer containing CMFSW, 10 mM EDTA, and a fast multienzyme cleaner (3M, Seoul, Republic of Korea). Using light microscopy, the tissues of *U. prolifera* were found to be intact, showing no visible lesions. To identify the seaweed species, we conducted DNA barcoding of the collected samples. For seaweed DNA extraction, we used DNeasy Plant Mini Kit (Qiagen, Hilden, Germany) as per the manufacturer’s instructions. We measured the quantity of the extracted DNA in both samples using Quant-IT PicoGreen (Invitrogen, Waltham, MA, USA).

### 2.3. Library Construction, Sequencing, and Adapter Trimming

For the DNA barcoding of seaweed, the nuclear-encoded internal transcribed spacers (ITS) regions ITS1(5′-CTTGGTCATTTAGAGGAAGTAA-3′)–ITS2(5′-GCTGCGTTCTTCATCGATGC-3′) and ITS3(5′-GCATCGATGAAGAACGCAGC-3′)–ITS4(5′-TCCTCCGCTTATTGATATGC-3′) were chosen for PCR amplification and automated sequencing. Seaweed bacteria sequencing libraries were prepared according to the Illumina 16S Metagenomic Sequencing Library procedures, emphasizing the V3(5′-CCTACGGGNGGCWGCAG-3′) and V4(5′-GACTACHVGGGTATCTAATCC-3′) regions. Herculase II fusion DNA polymerase (Agilent Technologies, Santa Clara, CA, USA) was used to amplify 2 ng input gDNA with 5× reaction buffer, dNTP mix, and universal F/R PCR primer. The initial PCR product was purified with AMPure beads (Agencourt Bioscience, Beverly, MA, USA), indexed for the final library construction with NexteraXT Indexed Primer, and quantified via qPCR. The second PCR was performed under the same conditions, except for being repeated 10 times. Paired-end sequencing was carried out on the MiSeq™ platform (Illumina, San Diego, CA, USA), following which Cutadapt (v3.2) was used to remove the adapter and primer sequences from the raw data [43].

The raw reads statistics and sequence quality evaluation from the primary analysis are shown in Table 1. The average percentage of ASVs was 92.58%. Every sample had paired-end fastq readings acquired. The BioSample accession numbers (SRR24893235, SRR24893234, SRR24893288, and SRR24893289) received for the project (PRJNA981356) are provided in Table 2 with details. The project was registered with GenBank.

### 2.4. Mineral Content Analysis

The mineral content was examined in 100 g samples of seaweed from Garorim Bay and Muan Bay. The feed standard analysis method was used to measure phosphorus and sulfur. Iodine measurements were taken using the ion meter method, while other components were measured using atomic absorption spectrophotometry.

### 2.5. Phylogenetic and Statistical Analysis

We aligned our ITS sequences with other *Ulva prolifera* ITS sequences from GenBank. Then, we used MEGA 11 to construct a phylogenetic tree with the maximum likelihood (ML) method and the Tamura-Nei model with 100 bootstrap replications [44,45]. We retrieved 16 *U. prolifera* ITS sequences from GenBank in September 2023. Sequences were aligned through Clustal W and, along with additional sequences we had obtained, were used to develop the tree for phylogenetic analysis. 

We attained the Amplicon Sequence Variants (ASVs) sequences through the processes of read error correction, merging, and denoising using DADA2 (v1.18.0) after each MiSeq run. We denoised the erroneous reads, merged the merged reads, and removed the chimera utilizing DADA2’s removeBimeraDenovo function. These refined ASVs were subsequently subjected to further analysis [46].

To obtain taxonomy information, we ran a BLAST+ (v2.9.0) search against the NCBI 16S Microbial Database for each ASV using the criteria (Query coverage > 85% and identity > 85%) [47]. Furthermore, QIIME (v1.9.0) was employed for downstream ASV analysis to ensure precise taxonomic designations and thorough data analysis. The Shannon and Simpson indices were calculated to assess the evenness and diversity of the microbial community. Further, we evaluated alpha diversity via Rarefaction curves and Chao1 values. Multiple alignments were executed using mafft (v7.475) and FastTreeMP (v2.1.10) [48].

All of the data were analyzed using a one-way ANOVA. Microsoft Excel 2016 MSO (16.0.4266.1001) was used to conduct the analysis. We conducted a Pearson correlation analysis between mineral contents and bacteria phyla from both bays. Tukey’s studentized range test (HSD (0.05)) was used to compare the means, and *p*-values less than 0.05 were deemed statistically significant. Lastly, we used OriginPro 2023b software to generate bar plots visualizing the differences. Open-source R software (ver. 4.3.1) was employed to produce colored heatmaps and conduct a principal component analysis (PCA) analysis.

PICRUSt2 was used to predict functional abundance through marker gene sequences [49]. Prior research employed PICRUSt2 analysis to forecast the metagenome of the microbial community using the Greengene database based on taxonomic abundance [50] and to predict the MetaCyc metabolic pathways of the sample microbiome [49,51]. To illustrate the microbiota’s function, ggplot (ver. 3.3.2) was used, and the Bray-Curtis distance was used to visualize how similar the clustering features were [52].

## 3. Results

### 3.1. Molecular Identification of Macroalgae

**Figure 3:**
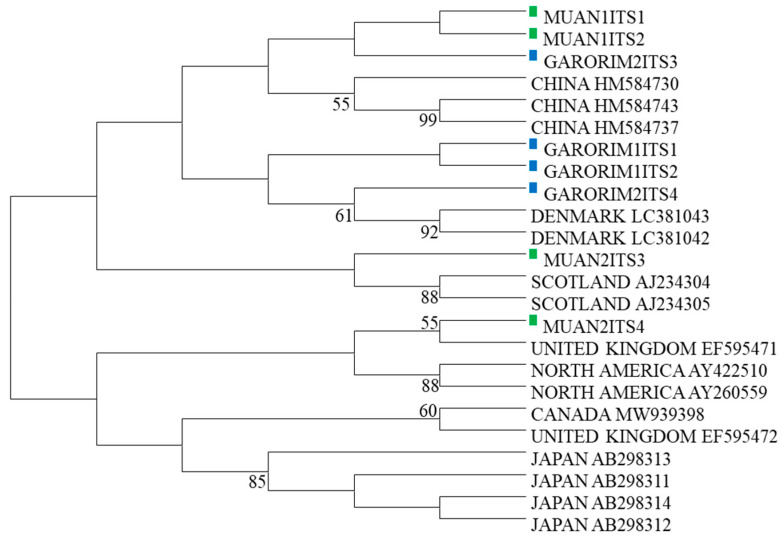
Phylogenetic tree based on ITS sequences. Sequences are labeled with the GenBank accession number of the ITS sequence and the taxon name.

Two samples were selected from the marine green macroalgae in Muan Bay and Garorim Bay, initially identified as *U. prolifera* based on their physical traits. The ITS1-2 and ITS3-4 regions of the *Ulva* species were sequenced, and accession numbers were obtained from GenBank (Table 2). A maximum likelihood tree was constructed using the ITS sequences, along with 16 other *Ulva* species’ sequences from GenBank (Table 3). The phylogenetic tree, which divided the *U. prolifera* branch of Republic of Korea into two groups, incorporated these ITS sequences (Figure 3). Samples GARORIM1ITS1, GARORIM2ITS2, GARORIM2ITS3, GARORIM2ITS4, MUAN1ITS1, and MUAN1ITS2 formed a distinct branch separate from the Japanese *U. prolifera* variants. However, samples MUAN2ITS3 and MUAN2ITS4 shared similarities with other outlier groups.

### 3.2. Mineral Analysis Results

Higher amounts of S, Ca, Mg, Na, and Fe were observed in the seaweed from Garorim Bay, as per the mineral analysis (Table 4). The S concentration in the seaweed of Garorim Bay amounted to 1913.83 ppm, while it was 1229.51 ppm in Muan Bay seaweed. The disparity in mineral content across different regions is attributed to varying degrees of freshwater inflow. For instance, Garorim Bay, a semi-closed bay, experiences minimal freshwater inflow. On the other hand, Muan Bay undergoes periodic freshwater influx from the nearby Yeongsan River [53], which notably impacts the sodium (Na) and sulfur (S) contents of its seaweed. Seaweeds are rich sources of polyphenols, polysaccharides, meroterpenoids, and terpenoids, and these bioactive molecules show potential for therapeutic drug discovery [54].

### 3.3. Epiphytic Bacterial Communities Diversity of Ulva prolifera

**Figure 4:**
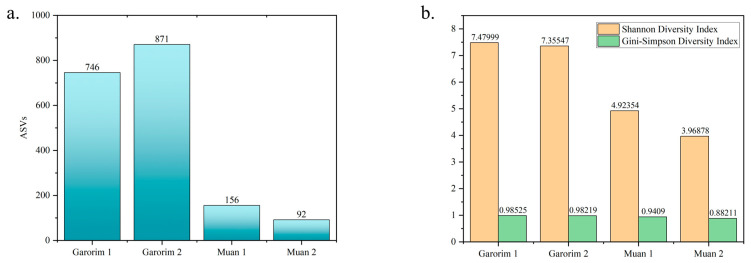
Diversity index between Garorim Bay and Muan Bay. (**a**) Number of different ASVs in the samples. (**b**) The alpha diversity of each sample was assessed using Shannon’s and Gini-Simpson’s diversity indexes.

**Figure 5:**
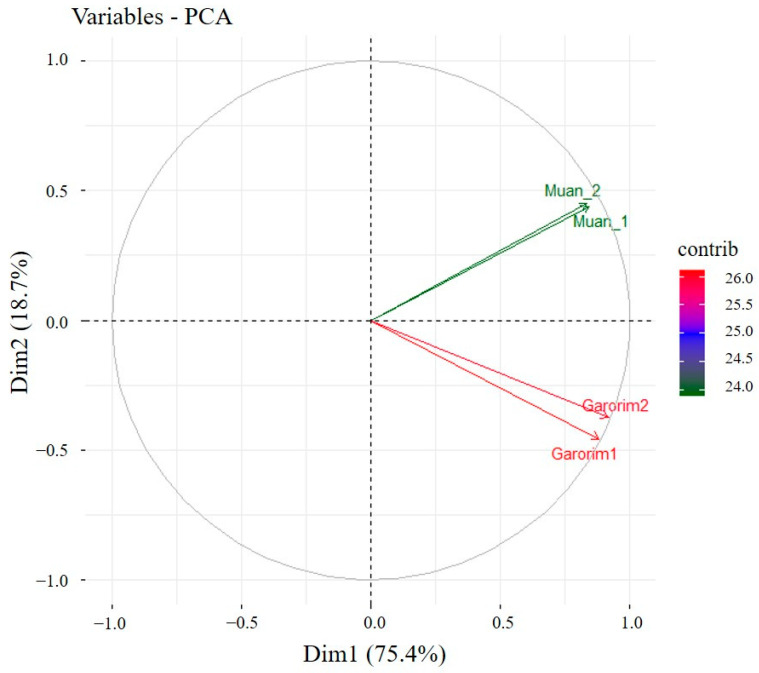
PCA (principal component analysis) of the seaweed samples from Garorim Bay and Muan Bay.

We utilized Shannon’s and Gini-Simpson’s diversity indices to examine each sample’s alpha diversity (Figure 4). The Simpson index reflects the number and evenness of species distribution in a sample. Significant disparities were highlighted by the Shannon indices between the samples (Figure 4). Garorim Bay seaweed demonstrated greater diversity (7.42 ± 0.06) than Muan Bay seaweed (4.45 ± 0.445). Regarding beta diversity, the PCA plot distinguished between the Muan Bay and Garorim Bay samples. PCA (Figure 5) illustrates a difference between Garorim Bay’s and Muan Bay’s bacterial communities. We also discovered the taxonomic diversity of *U. prolifera*-associated bacterial communities in both bays.

**Figure 6:**
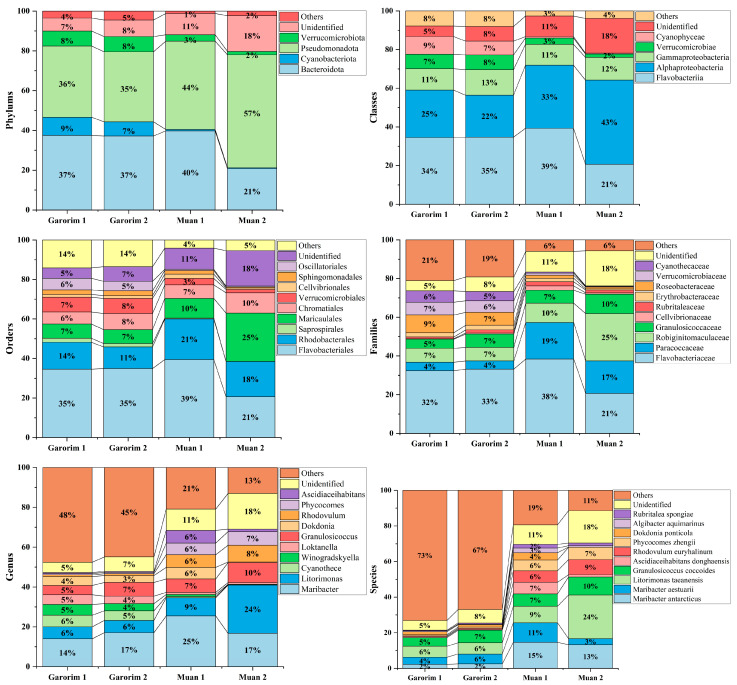
Relative abundance of bacterial ASVs at the phylum to species level. Taxonomic groups with a relative abundance lower than 1% were excluded from the plot legend flanking the bars.

**Figure 7:**
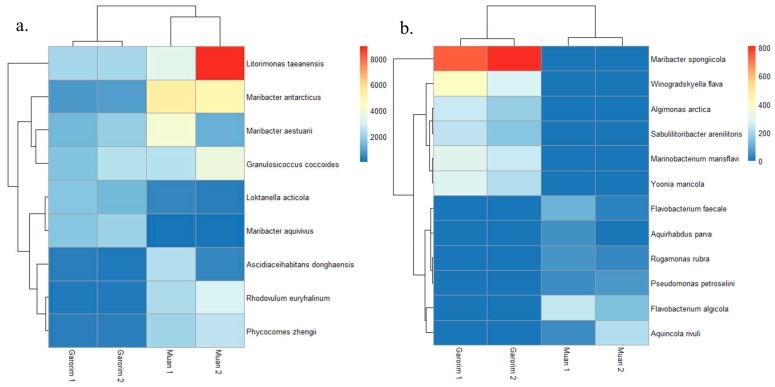
The abundance of bacterial species in Muan and Garorim seaweed. Red indicates high abundance, and blue indicates low abundance. (**a**) Differences between two samples; (**b**) Similarities between two samples.

In Muan Bay, the Pseudomonadota phylum is dominant, making up more than 50%. On the other hand, Garorim Bay’s leading microbiota consists of Pseudomonadota (35.5%), Bacteroidota (37%), Verrucomicrobia (8%), and Cyanobacteria (8%) (Figure 6). The Cyanobacteria percentage is relatively higher in Garorim Bay (8%) than in Muan Bay (0.5%). We observed a positive correlation of bacterial phyla (Bacteroidota, Cyanobacteria) with Ca, Mg, Na, and Fe, and a negative correlation of these minerals with the Pseudomonadota phylum (Table S1), whereas S was negatively correlated with Bacteroidota and Cyanobacteria and positively correlated with Pseudomonadota. Bacteria from the families *Flavobacteriaceae* (29.5%), *Paracoccaceae* (18%), *Robiginitomaculaceae* (17.5%), and *Granulosicococcaceae* (8.5%) had a higher taxonomy relative abundance ratio in Muan Bay. In Garorim Bay, *Flavobacteriaceae* (32.5%), *Robiginitomaculaceae* (7%)*, Granulosicococcaceae* (6%), two other families of *Roseobacteraceae* (8%), and *Verrucomicrobiaceae* (6.5%) were higher in relative abundance. *Maribacter aestuarii*, *Maribacter antarcticus*, and *Dokdonia ponticola* are significantly abundant Muan Bay seaweed species that are absent in the Garorim seaweed samples (Figure 7). On the contrary, *Maribacter aquivivus* is extremely abundant in Garorim Bay seaweed. *Maribacter aquivivus* significantly influences the bacterial community’s differentiation between Garorim Bay and Muan Bay seaweed samples. There are four species of Cyanobacteria found in Garorim Bay (*Pleurocapsa fuliginosa*, *Macrochaete psychrophila*, *Dulcicalothrix necridiiformans*, *Cyanothece aeruginosa*), of which *Cyanothece aeruginosa* is notably abundant (1930, 1648) (Figure S1).

### 3.4. Predictive Functional Roles of the Microbiome in U. prolifera

**Figure 8:**
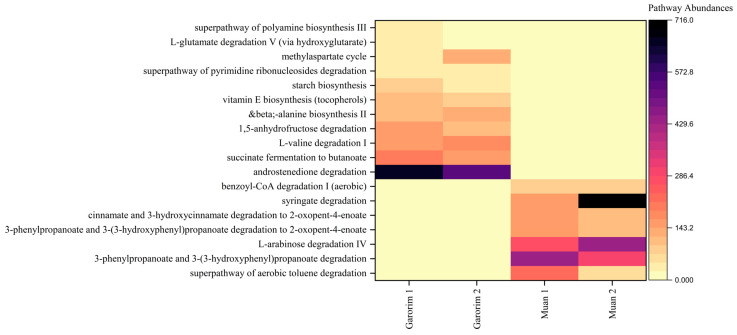
PICRUSt2 analysis of the abundant taxa yielding functional pathways derived from the macroalgae-associated microbiome samples analyzed in this study.

Heatmaps provided a visualization of the higher predicted metabolic potentials of the microbial community of each bay (Figure 8). Compared to the two sampling sites, only bacteria associated with Garorim Bay macroalgae showed abundant metabolic potentials related to the biosynthesis of starch, vitamin E, and beta-alanine, as well as the degradation of 1,5-anhydrofructose, L-valine, and androstenedione, and succinate fermentation. In contrast, Muan Bay displayed dominance in benzoyl-CoA, syringate, cinnamate and 3-hydroxycinnamate, 3-phenylpropanoate and 3-(3-hydroxyphenyl) propanoate, aerobic toluene super pathway, and L-arabinose degradation. Each pathway’s prevalence illustrates the metabolic potential of the macroalgal microbial community (Figure S2).

We categorized these metabolic functions into three groups. Group 1 consists of major biosynthetic pathways, including cell structure, cofactor, carrier, and vitamin biosynthesis, as well as the synthesis of fatty acids, lipids, carbohydrates, and amino acids. Garorim Bay pathways had a larger presence within these pathways. Nucleoside and nucleotide degradation, carbohydrate degradation, and amino acid degradation pathways were more common in Group 2. Lastly, Group 3 demonstrated a higher abundance of the tricarboxylic acid (TCA) cycle, glycolysis, and pyruvate-related fermentation pathways. The TCA cycle pathway was more prevalent in Muan Bay, whereas Garorim Bay had greater representation within the other pathways.

**Figure 9:**
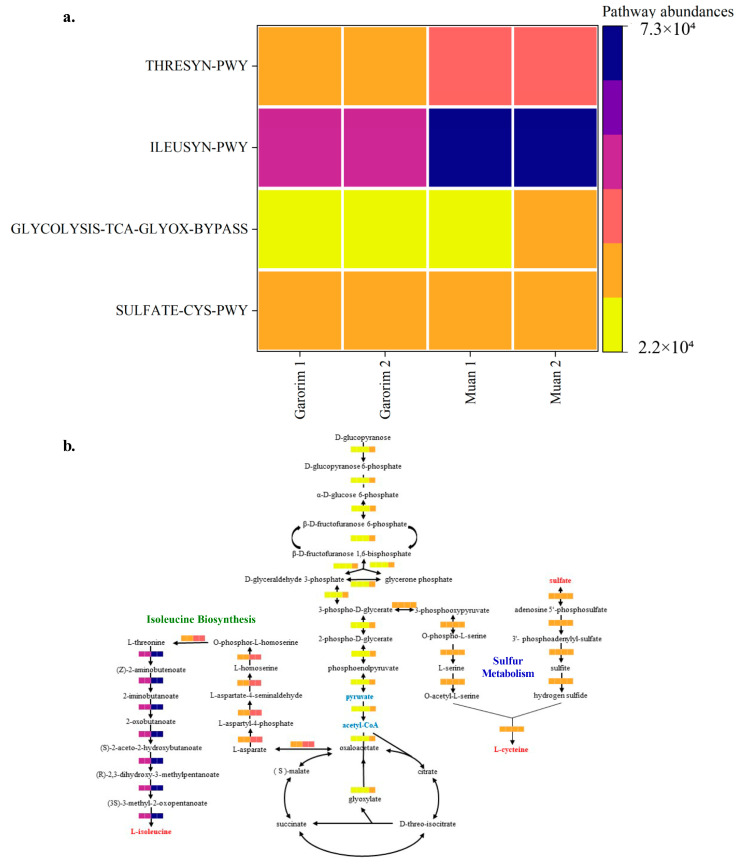
PICRUSt2 analysis of the abundant taxa yielding functional pathways derived from the microbiome samples analyzed in this study. (**a**) Heatmap of the selected pathway abundances. (**b**) The putative functions of the microbiota are presented as heatmaps through PICRUSt2 analysis. The pathways were constructed based on the MetaCyc database (GLYCOLYSIS-TCA-GLYOX-BYPASS, THRESYN-PWY, ILEUSYN-PWY, and SULFATE-CYS-PWY).

Hierarchical clustering was executed using the R program to identify sample groups with related functional pathways (Figure 9). It is plausible that the abundant GLYCOLYSIS-TCA-GLYOX-BYPASS, THRESYN-PWY, and ILEUSYN-PWY pathways in Muan Bay indicate high metabolic activity and elevated energy production. This is achieved through glycolysis, the TCA cycle, and the biosynthesis of threonine and isoleucine within the microbiome community. Conversely, a lower prevalence of these pathways may suggest varying environmental conditions and nutrient availability. Furthermore, in situations of stress and dormancy, microorganisms tend to reduce metabolic activity to conserve energy and resources. It is also worth noting that the microorganisms associated with the SULFATE-CYS-PWY pathway could potentially play a role in sulfate assimilation and cysteine biosynthesis.

## 4. Discussion

Identifying different *Ulva* species is a challenging task in phycology due to their basic morphology and considerable intraspecific variation in the few morphological features used for species characterization [55,56,57]. Morphological identification of *Ulva prolifera* in Republic of Korea has been conducted before, but no molecular identification using ITS1-2 and ITS3-4 primers has been performed. Some studies have suggested that the phenotypic traits of the *Ulvaceae* family are unstable and that the family’s relatively simple morphological features may lead to phenotypic overlaps among various *Ulvaceae* species [58]. As a result, we studied green marine seaweed (identified as *Ulva prolifera*), compared intraspecific genetic variations with *Ulva prolifera* from other countries, and opted not to use the 5s rDNA spacer as a marker due to its lack of effectiveness in distinguishing different geographical populations of *Ulva prolifera*. Instead, we utilized the ITS of the ribosomal RNA gene to assist with *U. prolifera* species identification. Our research revealed a high genetic variation within *U. prolifera*, leading to the identification of unique variants differing from Chinese and Japanese *Ulva prolifera*. Moreover, the ITS 4 marker sequences of both Garorim Bay and Muan Bay seaweed showed similarities with the United Kingdom’s variants, indicating a significant intraspecific genetic variability within *U. prolifera*.

We analyzed the alpha (single host diversity) and beta (differing host diversity) diversity in Garorim Bay and Muan Bay’s two marine seaweeds through 16S rRNA gene sequencing. Alpha diversity, as indicated by the Shannon and Gini-Simpson indexes, showed a diverse community in the bacteria associated with *Ulva prolifera* in Garorim Bay. In Garorim Bay, the *U. prolifera* library had 521 species (Chao1 estimate 811.09) across 15 phyla, notably more than Muan Bay’s 143 species (Chao1 estimate 124) across 12 phyla. Nonparametric Chao1 index data (747.04, 875.14) showed that Garorim Bay had the richest bacterial community, whereas the figures were considerably lower in Muan Bay (156, 92). We hypothesize that variations in mineral content could be a possible reason for the numbers of ASVs in both samples. Four minerals (Ca, Mg, Na, and Fe) and the bacterial phyla Cyanobacteria showed a highly positive correlation (r^2^ = 0.98206, *p*-value = 0.01794; r^2^ = 0.98267, *p*-value = 0.01733; r^2^ = 0.98235, *p*-value = 0.01765; r^2^ = 0.98241, *p*-value = 0.01759) (Table S1). Moreover, two more phyla (Bacteroidota and Verrucomicrobia) were positively correlated with these four minerals. We found that all of these phyla (Bacteroidota, Cyanobacteria, and Verrucomicrobia) were comparatively higher in Garorim Bay seaweed samples. Beta diversity analyses revealed no overlap between the Garorim Bay and Muan Bay samples. This was confirmed by PCA analysis, highlighting differences in Garorim and Muan Bay’s microbial communities (Figure 5). In our study, although Pseudomonadota and Bacteroidota were common, Pseudomonadota accounted for more than 50% of the samples from Muan Bay. Conversely, the availability of Cyanobacteria and Verrucomicrobia in Muan Bay (0.5%, 2.5%) differed from that in Garorim Bay (8%, 8%), possibly explaining the absence of community overlap.

The 16S rRNA gene sequences produced by epiphytic bacteria from the green alga *Enteromorpha* sp. reveal a prevalence of Gammaproteobacteria and Bacteroidota members [59]. *Ulva rigida*, a Spanish green alga, hosts the flavobacterium group [60], while Plantomycetes was found in another *Ulva* species from Portugal [61]. A study on *Ulva australis* revealed that Deltaproteobacteria and Actinobacteria are the most abundant species [62]. In our study, Pseudomonadota and Bacteroidota were the most abundant phyla in both bays. Burke’s metagenomic analysis of bacterial communities associated with *U. australis* also found that Pseudomonadota (64%), Bacteroidota (27.6%), and Planctomycetes (3.4%) represented the most prevalent taxa [37]. In Muan Bay, Pseudomonadota was the predominant active group, and it was second in abundance in Garorim Bay, with most sequences belonging to the Gammaproteobacteria and Alphaproteobacteria classes. Heatmaps showed the most abundant species of the bays, where species from Pseudomonadota (*Litorimonas taeanensis* and *Granulosicoccus coccoides*) and Bacteroidota (*Maribacter antarcticus* and *Maribacter aestuarii*) indicated higher abundance (Figure 7a). The genus *Maribacter* can produce auxin, which may affect the enlargement of newly divided algal cells [32]. Moreover, recent studies have found that *Maribacter* spp. help in the development of rhizoid cells and cell walls [24]. Notably, Gammaproteobacteria *Granulosicoccus coccoides* showed a high prevalence in our data, mainly from Muan Bay (Figure 6). Previous reports have indicated frequent associations between this genus and marine macroalgae [32]. Various macroalgae, including *Saccharina japonica* [63], *Fucus vesiculosus* [64], and *Porphyra umbilicalis* [65], host bacterial communities primarily consisting of the *Granulosicoccus* genus. There is scope for studying the interactions of these species and *U. prolifera*.

Cyanobacteria play a crucial role in nitrogen fixation in the tropical marine ecosystem, as stated by Hoffman [66]. For example, nitrogen-fixing Cyanobacteria constitute a significant part of the microbial community linked with *L. dendroidea* [67]. Philips and Zeman reported the nitrogen-fixing activity of Oscillatoria in tandem with the *Sargassum* thalli [68]. The most prevalent Cyanobacteria species, *Cyanothece aeruginosa*, together with *Lyngbya* and *Synechocystis*, were identified as depending on the temporal separation between photosynthesis and nitrogen fixation, primarily at night [69,70]. Our data included cyanobacterial species from different orders (Nostocales, Oscillatoriales, Synechococcale), with *Cyanothece aeruginosa*, *Pleurocapsa fuliginosa*, *Dulcicalothrix necridiiformans*, and *Macrochaete psychrophila* abundant in Garorim Bay. These cyanobacterial species might interact with *U. prolifera* in Garorim Bay, contributing to their nitrogen fixation, nutrient recycling, and enhanced growth in marine ecosystems. Garorim Bay had higher sulfur levels than Muan Bay (Table 4). Investigations have revealed the integral role of sulfur assimilation in Cyanobacteria and its influence on other cellular functions [71]. The Cyanobacteria genus *Synechococcus* can derive sulfur from certain sources. In our study, however, *Synechococcus mooriganga* was barely present in our data. 

Our study reveals that the epiphytic microbiomes of the Garorim and Muan groups, analyzed using PICRUSt2, predict their associated metabolic potentials (Figure S2). Both samples displayed abundant metabolic potential in the microbial communities. We noted a higher engagement in biosynthesis pathways concerning amino acids, nucleosides, nucleotides, cofactors, carriers, vitamins, fatty acids, lipids, and carbohydrates. The bacterial community in Garorim Bay exhibited the predicted metabolic potentials of succinate fermentation, androstenedione degradation, and L-valine. In Muan Bay, the bacterial community had predicted metabolic potentials related to 3-(3-hydroxyphenyl) propanoate, L-arabinose (Figure 8). Enzyme cofactors play a crucial role in microbial metabolism [72], while vitamins, notably vitamin B12, aid algal hosts [73]. The superpathway of glycolysis, pyruvate dehydrogenase, TCA, and glyoxylate bypass (GLYCOLYSIS-TCA-GLYOX-BYPASS) provides energy for synthesizing sugar, protein, and other substances, as is evident in Muan and Garorim Bays [74] (Figure 9). According to PICRUSt2 metabolite analysis, Muan Bay showed higher isoleucine and threonine biosynthesis, potentially due to environmental differences.

Nitrogen, phosphorus, and iron are usually the most limiting nutrients for cyanobacterial growth [75,76]. Phosphorus structures and functions in various metabolic processes, influencing respiration and photosynthesis, along with ATP-dependent enzyme activities [77,78]. Nitrogen shortage can delay cell growth and instigate chlorosis, a condition that degrades photosynthetic pigment and may eventually downregulate photosynthesis [14,15]. Iron is needed for growth and photosynthesis, but needs to be balanced to protect against oxidative stress [79,80]. Levels of S, Ca, Mg, NA, Fe, Cu, and Mn were found to be higher in Garorim Bay. The nutrient requirements for nitrogen-fixing Cyanobacteria may differ from those of their non-nitrogen-fixing counterparts [81]. The differing mineral contents in Garorim and Muan Bays likely reflect their distinct environmental conditions.

## 5. Conclusions

Our study aimed to characterize the alpha and beta diversity of bacterial communities linked to the marine green seaweed *Ulva prolifera* from Garorim Bay and Muan Bay. We used 16S rRNA gene sequencing for this study. Garorim Bay exhibited a higher alpha diversity based on the Shannon diversity index. Meanwhile, beta diversity analysis showed distinct microbial communities in each bay. These differences were significant according to PCA. We noted variations in specific bacterial phyla between the bays. Muan Bay had a higher abundance of Pseudomonadota, while Cyanobacteria and Verrucomicrobia were more common in Garorim Bay. Bacteria from the families *Flavobacteriaceae*, *Robiginitomaculaceae*, and *Granulosicoccaceae* had notable abundance in both bays. Further investigation revealed differences in the predicted metabolic potentials of the microbial communities. We also considered environmental factors, like salinity and nutrient availability, which could be impacting the variations in the two bays’ microbiomes. Our results offer important insights into the microbial community diversity linked to *U. prolifera*, underlining the importance of specific bacterial taxa and metabolic potentials in marine environments. Further studies can look into the connection between Cyanobacteria and sulfur content in Garorim Bay’s *U. prolifera* population.

## Figures and Tables

**Table 1:** Raw reads statistics and sequence quality assessment of 16S rRNA sequences from the *Ulva prolifera* epiphytic bacterial communities.

Sample ID	Total Bases (bp) ^a^	Total Reads ^b^	GC (%) ^c^	AT (%) ^d^	Q20 (%) ^e^	Q30 (%) ^f^
Garorim 1	48,043,814	159,614	51.3	48.7	93.3	86.2
Garorim 2	54,293,778	180,378	51.2	48.8	94	87.2
Muan 1	46,788,644	155,444	51.2	48.8	94.9	88.9
Muan 2	46,719,414	155,214	51	49	95	88.9

^a^ Total bases (bp): Total number of bases sequenced. ^b^ Total reads: Total number of reads. For Illumina paired-end sequencing, this value refers to the sum of read1 and read2. ^c^ GC (%): Ratio of GC content. ^d^ AT (%): Ratio of AT content. ^e^ Q20 (%): Ratio of bases that have a phred quality score of over 20. ^f^ Q30 (%): Ratio of bases that have a phred quality score of over 30.

**Table 2:** Identical numbers (Accession numbers) of the macroalgae samples obtained from GenBank from DNA barcoding sequences.

Sample Name	Scientific Name	Accession Number
Garorim ITS1-2	*Ulva prolifera*	SRR24893235
Garorim ITS3-4	*Ulva prolifera*	SRR24893234
Muan ITS1-2	*Ulva prolifera*	SRR24893288
Muan ITS3-4	*Ulva prolifera*	SRR24893289

**Table 3:** Specimens of *Ulva prolifera* used in this study were collected from GenBank.

Serial Numbers	Locations	Accession Numbers	Primers
1.	Japan	AB298314	ITS
2.	Japan	AB298312	ITS
3.	Japan	AB298311	ITS
4.	Japan	AB298313	ITS
5.	China	HM584730	ITS
6.	China	HM584743	ITS
7.	China	HM584737	ITS
8.	Scotland	AJ234304	ITS
9.	Scotland	AJ234305	ITS
10.	United Kingdom	EF595471	ITS
11.	United Kingdom	EF595472	ITS
12.	North America	AY422510	ITS
13.	North America	AY260559	ITS
14.	Denmark	LC381043	ITS
15.	Denmark	LC381042	ITS
16.	Canada	MW939398	ITS

**Table 4:** Mineral composition of *Ulva prolifera* collected from Garorim Bay and Muan Bay.

Mineral	Garorim Bay (ppm)	Muan Bay (ppm)
S	1913.83	1229.51
Ca	1523.68	969.95
Mg	2088.80	811.13
Na	1541.99	405.40
Fe	911.25	85.52
K	644.14	753.59
Zn	8.15	8.20
Cu	8.91	2.05
Mn	67.80	5.23
P	118.07	161.77
I	29.38	33.48

## Data Availability

The original data presented in the study are openly available in [NCBI Sequence Read Archive (SRA) database] at [accession number PRJNA981356]. The raw data supporting the conclusions of this article will be made available by the authors on request.

## Supplementary Materials

The following supporting information can be downloaded at: https://www.mdpi.com/article/10.3390/microorganisms12061142/s1, Figure S1: Relative abundance of Cyanobacteria at the species level of the green seaweed *Ulva prolifera* in Garorim Bay and Muan Bay. Figure S2: Different functional pathways derived from Garorim Bay and Muan Bay; Table S1: Pearson correlation between mineral content and bacterial phyla of the microbial community of *Ulva prolifera*.
