# Supplementary material for: Epiphytic Bacterial Community Analysis of Ulva prolifera in Garorim and Muan Bays, Republic of Korea
Source: Microorganisms. 2024 Jun 4;12(6):1142. doi: 10.3390/microorganisms12061142 (PMC11205692; doi:10.3390/microorganisms12061142)
Supplement: Supplementary file 1 [file microorganisms-12-01142-s001.zip › Table S1.pdf]

Table S1: Pearson correlation between mineral content and bacterial phyla of the microbial community of *Ulva prolifera*.

		S	Ca	Mg	Na	Fe	K	P	I	Bacteroidota	Cyanobacteriota	Planctomycetota	Pseudomonadota	Verrucomicrobiota
S	Pearson Corr.	1	-0.52198	-0.52445	-0.52315	-0.52342	0.53723	0.55328	0.7367	-0.99742*	-0.53547	-0.54592	0.88138	-0.69061
	p-value	--	0.47802	0.47555	0.47685	0.47658	0.46277	0.44672	0.2633	0.00258	0.46453	0.45408	0.11862	0.30939
Ca	Pearson Corr.	-0.52198	1	0.99999*	1*	1*	-0.99968*	-0.9986*	-0.89558	0.46031	0.98206*	0.95458*	-0.86259	0.9773*
	p-value	0.47802	--	<0.0001	<0.0001	<0.0001	3.25E-04	0.0014	0.10442	0.53969	0.01794	0.04542	0.13741	0.0227
Mg	Pearson Corr.	-0.52445	0.99999*	1	1*	1*	-0.99977*	-0.99881*	-0.89739	0.46292	0.98267*	0.95389*	-0.86399	0.97787*
	p-value	0.47555	<0.0001	--	<0.0001	<0.0001	2.29E-04	0.00119	0.10261	0.53708	0.01733	0.04611	0.13601	0.02213
Na	Pearson Corr.	-0.52315	1*	1*	1	1*	-0.99972*	-0.9987*	-0.89644	0.46155	0.98235*	0.95425*	-0.86326	0.97757*
	p-value	0.47685	<0.0001	<0.0001	--	<0.0001	2.77E-04	0.0013	0.10356	0.53845	0.01765	0.04575	0.13674	0.02243
Fe	Pearson Corr.	-0.52342	1*	1*	1*	1	-0.99973*	-0.99872*	-0.89663	0.46183	0.98241*	0.95418*	-0.86341	0.97764*
	p-value	0.47658	<0.0001	<0.0001	<0.0001	--	2.67E-04	0.00128	0.10337	0.53817	0.01759	0.04582	0.13659	0.02236
K	Pearson Corr.	0.53723	-0.99968*	-0.99977*	-0.99972*	-0.99973*	1	0.99962*	0.90663	-0.47644	-0.98558*	-0.95002*	0.87107	-0.98062*
	p-value	0.46277	3.25E-04	2.29E-04	2.77E-04	2.67E-04	--	3.77E-04	0.09337	0.52356	0.01442	0.04998	0.12893	0.01938
P	Pearson Corr.	0.55328	-0.9986*	-0.99881*	-0.9987*	-0.99872*	0.99962*	1	0.91787	-0.49347	-0.98865*	-0.94442	0.87956	-0.98348*
	p-value	0.44672	0.0014	0.00119	0.0013	0.00128	3.77E-04	--	0.08213	0.50653	0.01135	0.05558	0.12044	0.01652
I	Pearson Corr.	0.7367	-0.89558	-0.89739	-0.89644	-0.89663	0.90663	0.91787	1	-0.69635	-0.9465	-0.78076	0.92531	-0.9387
	p-value	0.2633	0.10442	0.10261	0.10356	0.10337	0.09337	0.08213	--	0.30365	0.0535	0.21924	0.07469	0.0613
Bacteroidota	Pearson Corr.	-0.99742*	0.46031	0.46292	0.46155	0.46183	-0.47644	-0.49347	-0.69635	1	0.47818	0.48532	-0.84546	0.63756
	p-value	0.00258	0.53969	0.53708	0.53845	0.53817	0.52356	0.50653	0.30365	--	0.52182	0.51468	0.15454	0.36244

Cyanobacteriota	Pearson Corr.	-0.53547	0.98206*	0.98267*	0.98235*	0.98241*	-0.98558*	-0.98865*	-0.9465	0.47818	1	0.88432	-0.85665	0.96345*
	p-value	0.46453	0.01794	0.01733	0.01765	0.01759	0.01442	0.01135	0.0535	0.52182	--	0.11568	0.14335	0.03655
Planctomycetota	Pearson Corr.	-0.54592	0.95458*	0.95389*	0.95425*	0.95418*	-0.95002*	-0.94442	-0.78076	0.48532	0.88432	1	-0.85794	0.94784
	p-value	0.45408	0.04542	0.04611	0.04575	0.04582	0.04998	0.05558	0.21924	0.51468	0.11568	--	0.14206	0.05216
Pseudomonadota	Pearson Corr.	0.88138	-0.86259	-0.86399	-0.86326	-0.86341	0.87107	0.87956	0.92531	-0.84546	-0.85665	-0.85794	1	-0.95019*
	p-value	0.11862	0.13741	0.13601	0.13674	0.13659	0.12893	0.12044	0.07469	0.15454	0.14335	0.14206	--	0.04981
Verrucomicrobiota	Pearson Corr.	-0.69061	0.9773*	0.97787*	0.97757*	0.97764*	-0.98062*	-0.98348*	-0.9387	0.63756	0.96345*	0.94784	-0.95019*	1
	p-value	0.30939	0.0227	0.02213	0.02243	0.02236	0.01938	0.01652	0.0613	0.36244	0.03655	0.05216	0.04981	--
