# Supplementary figures and images for: Epiphytic Bacterial Community Analysis of Ulva prolifera in Garorim and Muan Bays, Republic of Korea
Source: Microorganisms. 2024 Jun 4;12(6):1142. doi: 10.3390/microorganisms12061142 (PMC11205692; doi:10.3390/microorganisms12061142)

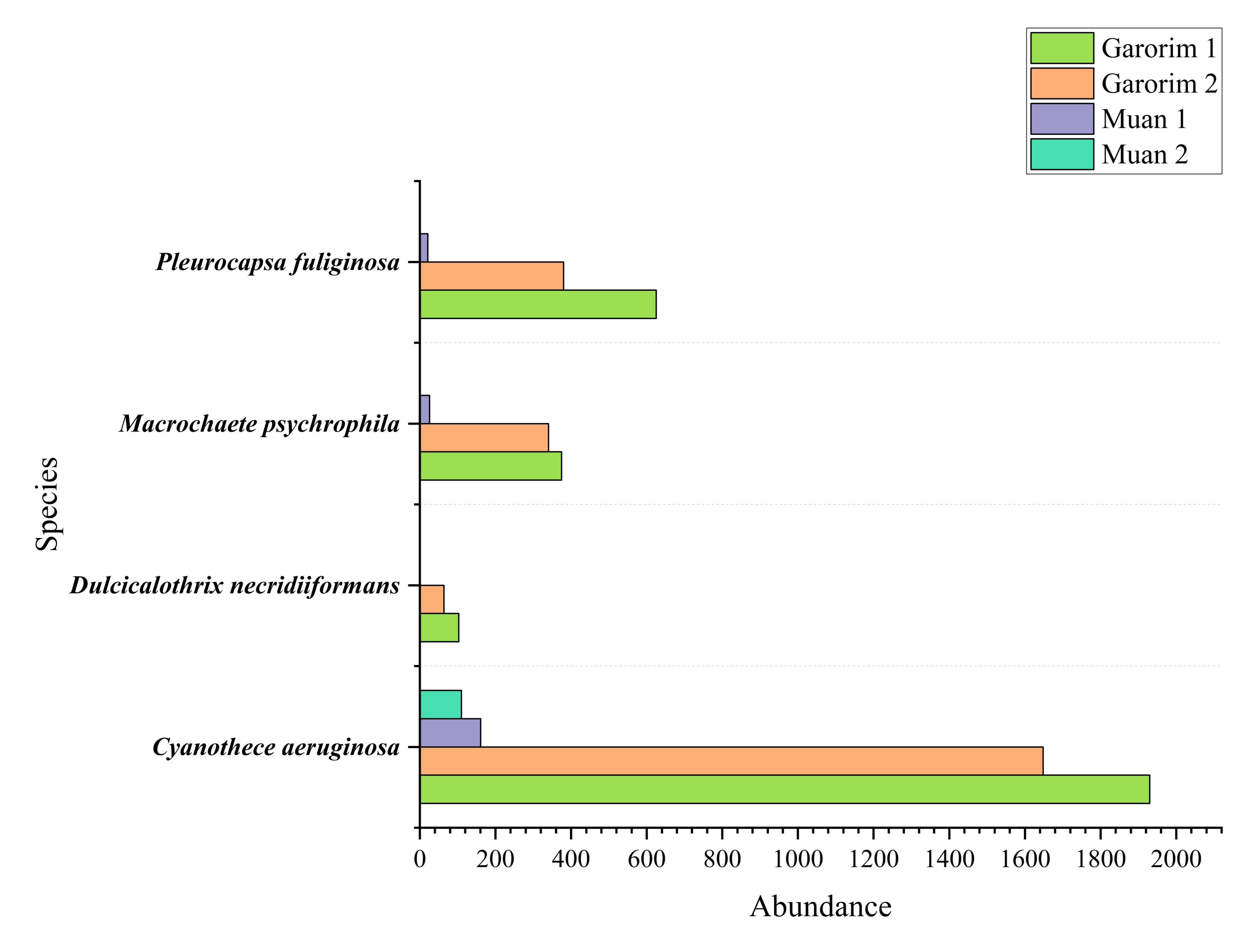

Supplement: Supplementary file 1 [file microorganisms-12-01142-s001.zip › Figure S1.tif]

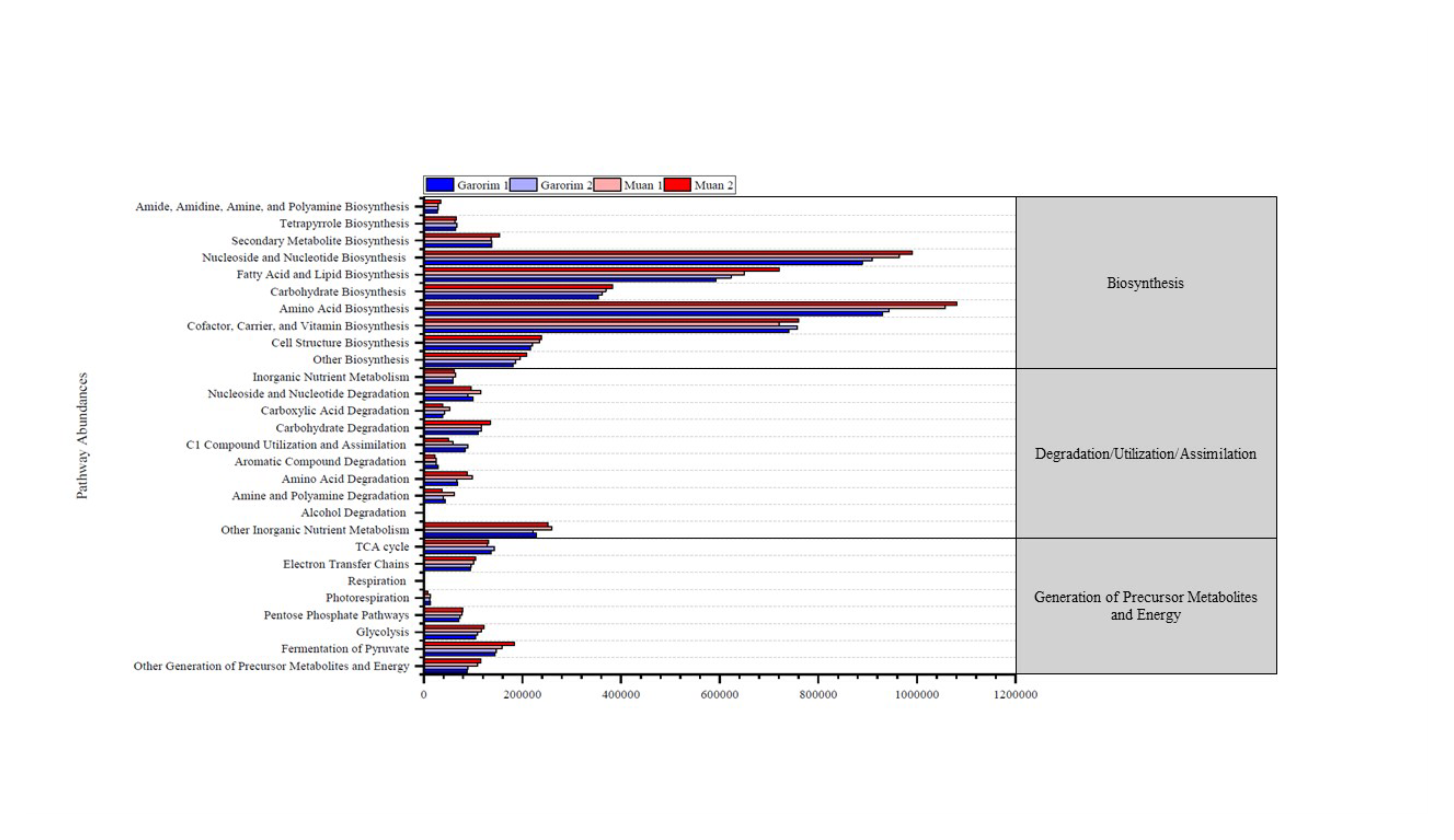

Supplement: Supplementary file 1 [file microorganisms-12-01142-s001.zip › Figure S2.tif]
